# Deciphering and reconstitution of positional information in the human brain development

**DOI:** 10.1186/s13619-021-00091-7

**Published:** 2021-09-01

**Authors:** Yi-Fan Wang, Cong Liu, Peng-Fei Xu

**Affiliations:** 1 Women’s Hospital, and Institute of Genetics, Zhejiang University School of Medicine, Hangzhou, Zhejiang China; 2 Institute of Zhejiang University and University of Edinburgh, Jiaxing, Zhejiang China; 3 Department of Biochemistry, Yong Loo Lin School of Medicine, National University of Singapore, 14 Medical Dr, Singapore, 117599 Singapore

## Abstract

Organoid has become a novel in vitro model to research human development and relevant disorders in recent years. With many improvements on the culture protocols, current brain organoids could self-organize into a complicated three-dimensional organization that mimics most of the features of the real human brain at the molecular, cellular, and further physiological level. However, lacking positional information, an important characteristic conveyed by gradients of signaling molecules called morphogens, leads to the deficiency of spatiotemporally regulated cell arrangements and cell–cell interactions in the brain organoid development. In this review, we will overview the role of morphogen both in the vertebrate neural development in vivo as well as the brain organoid culture in vitro*,* the strategies to apply morphogen concentration gradients in the organoid system and future perspectives of the brain organoid technology.

## Background

Understanding human brain development and relevant neurological diseases have been one of the most important challenges in current biology. Considering human ethics, it is difficult to directly work on the live human brains. The brain specimens taken from the corpses cannot be sufficient to examine the dynamic development and function of the brain, therefore, animal models such as mice are widely used. However, there are a lot of nonnegligible differences between the brain of animal models and humans. For example, the surface of the mouse brain is smoother, while the neocortex is largely expanded and highly folded in humans due to the contribution of ventricular radial glial cells (vRGCs) and outer radial glial cells (oRGCs) (Lui et al. 2011). Besides, the division patterns of neural stem cells varied and become more complicated in humans, rodents are born with a relatively intact brain with most of its neurons in it, whereas human children do not develop large numbers of neurons in the cerebral cortex until after birth (Homem et al. 2015). These obstacles make it hard to dissect and comprehend the developmental process of the human brain as well as the relevant neurodevelopmental and neurodegenerative diseases detailly. Therefore, a better model system that can recapitulate the features and functions of the human brain is urgently required for promoting research in this field.

The organoid is a novel technique with rapid development in this century. It is a self-organized structure with multiple organ-specific cell types that is able to recapitulate the specific function of the corresponding organs (Lancaster and Knoblich 2014). Currently, organoids are often derived from embryonic stem cells, organ progenitor cells or induced pluripotent stem cells (iPSCs). Existing types including cerebral, gut, hepatic, pancreatic, epithelial organoid and so on are widely used in researching both developmental biology and diseases (Lancaster et al. 2013; Sato et al. 2009; Huch et al. 2015; Barker et al. 2007). For brain organoids, the intact systems of hPSC-derived neural organoids have been built since the early 2010s (Lancaster et al. 2013). These systems well reproduce the developmental processes and organization of the developing human brain. In addition, it transformed the traditional 2D tissue culture techniques to more complicated 3D cell culture systems which can better mimic in vivo environments. This fancy technique provides an important supplement to investigate those events that only occur in humans. However, although the current organoid can recapitulate most of the molecular and cellular features, lacking a reproducible topographic organization (i.e. each cell type and extracellular matrices are located in their corresponding sites along body axes) has become an intractable challenge for the development of the whole organoid technique. In this case, establishing a more complete organoid culture system with positional information might be one main direction in organoid research.

Early in 1885, Hans Driesch had already proposed a coordinate system that specified the position of each developmental cell, based on his experiments on sea urchin embryo: separated two cells of the embryo can develop into a complete sea urchin in a correct manner, although with a smaller size (Wolpert 2016). However, for the first time that positional information (PI) emerged, it should be traced back to the “French Flag” model presented by Lewis Wolpert in 1969 (Wolpert 1969). In this model, an idealized morphogen signal is raised to interpret the cells’ position and further spatial patterns that drive larger-scale morphogenesis from a molecular perspective. These morphogens are thought to be emanated from a localized source and can diffuse to establish a concentration gradient and create a biochemical boundary for controlling cell proliferation, differentiation and migration. Cells respond to these signals for further fate and positional determination by inducing different target genes at different exposing duration and amounts. During the past 50 years, quantities of morphogens are identified to not only specify the cell fate but also provide the positional information to the organ or the whole embryo to form a complicated 3D organization. The formation of the vertebrate neural tube, the embryonic precursor to the central nervous system, is quite an elaborate process highly requested for the correct specification and positional information. A number of morphogens have been proved to participate in the development of the neural tube. Retinoic acid (RA), Fibroblast Growth Factors (FGF), Growth Differentiation Factors (GDF) are involved in the anterior–posterior patterning, Wingless-Int (Wnt), Bone morphogenetic proteins (BMPs) and Sonic Hedgehog (Shh) signaling are the most important known morphogens that function on neural tube dorsal–ventral axis patterning (Le Dréau and Martí 2012).

In this article, starting from the summary of brain organoid research history and current progress, we will review and discuss the importance of the role of three morphogens, Wnt, BMP and Shh both in brain development in vivo as well as the brain organoid culture in vitro*.* We will also summarize current strategies to set up positional information in the brain organoid culture system to provide some future perspectives for the whole organoid research field.

## Main Text

### The history of human brain organoid research and current progress

**Fig. 1:**
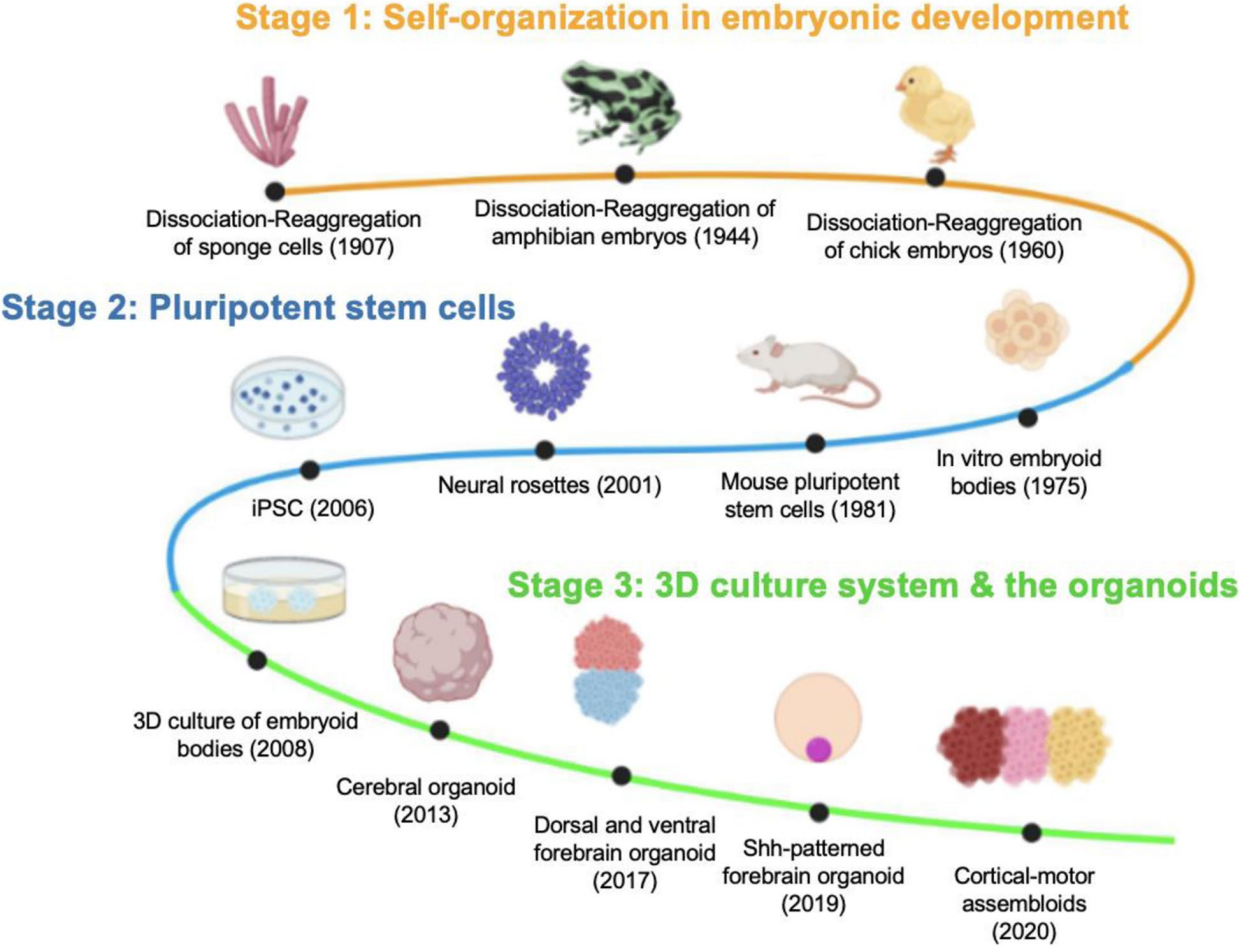
History of 3D brain organoid culture system development. The history of 3D brain organoid research here can be traced back to the early twentieth century and divided into three main stages: (1) Self-organization in embryonic development: The previous biologist has done a series of anatomical experiments to dissociate cells from the sponge (Wilson 1907), amphibian embryos (Tung and Kü 1944) and chick embryos (Zwilling 1960) and reaggregate to form the corresponding structures, pointing out the key role of self-organization in the embryonic development; (2) Pluripotent stem cells: differentiation system after the formation of in vitro embryoid bodies (Martin and Evans 1975) and the generation of pluripotent stem cells (Martin 1981; Takahashi and Yamanaka 2006) has established the basis for the organoid technology. Using these systems, a group of polarized neural progenitor cells that resemble the early neural tube could be generated; (3) 3D culture system & the organoids: with the PSC, scientists have developed 3D culture system instead of an original 2D culture system to generate a better human in vitro model called the organoids. Using different culture strategies, different region-specific brain organoids were generated to mimic human brain development

Embryonic development is a robust and complicated process that is orchestrated by multiple regulatory mechanisms at different scales of the organization. With these intrinsic and spontaneous capacities, embryonic stem cells could automatically self-organize to generate the correct three-dimensional and functional structures (Werner et al. 2017). Early in 1907, Wilson has identified the self-organization ability through dissociation and reaggregation of sponge cells (Wilson 1907). Later, this ability has been expanded to more complicated organisms such as amphibians and chicks (Tung and Kü 1944; Zwilling 1960). These anatomical researches have also settled the theoretical and technical basis for the further exploration of the in vitro culture system (Fig. 1).

In 1975, Martin and Evans have successfully formed an embryoid body using isolated single teratocarcinoma cells that can differentiate into almost all of the cell types in vitro, which can be regarded as the beginning of the in vitro culture system. Since then, the advance in pluripotent stem cell research including embryonic stem cell (Thomson et al. 1998) and iPSCs (Takahashi and Yamanaka 2006) has widely expanded the sources and the differentiation potential of the embryoid bodies. In 2001, Zhang et al. have successfully used such culture systems to generate neural progenitor cells with the apical-basal polarity that can resemble early neural tube structures*.* However, these protocols were still based on the 2D culture system (i.e., monolayer culture system), therefore, there are still some challengeable limitations for modeling complicated human structures such as the brain. For example, the concentration gradients of different growth factors and morphogens, which are important for the cell fate decision and regional specification in human brain development, are hard to completely recapitulate in the limited monolayer system. Besides, the cell–cell interactions and cell migration during neurogenesis would be easily lost in such systems.

Thanks to these considerations of 2D culture protocols, the 3D culture systems have emerged in this century. One early representative method is the serum-free culture of embryoid-like aggregate culture with quick reaggregation (SFEBq) developed by Sasai Lab (Eiraku et al. 2008). Using this efficient three-dimensional aggregation culture system, the mouse cortex (Eiraku et al. 2008) and human retinal tissue (Eiraku et al. 2011) were successfully recapitulated in vitro. Moreover, this study also confirms that embryonic stem cells (ESC) derived tissues could self-organize to resemble human embryonic corticogenesis (Eiraku et al. 2008). Later in 2013, Lancaster et al. have published a protocol to generate cerebral organoids with the assistance of Matrigel matrix and spinning bioreactor. The brain organoids generated by this system can well reproduce the developmental processes and organization of the developing human brain. Compared to previous protocols, some human-specific features such as enlarged outer subventricular zone, division patterns of neural stem cells and neuronal migration can be well recapitulated (Lancaster et al. 2013).

In the following years, various approaches to generate human brain organoids were emerged to achieve higher efficiency and accuracy to recapitulate different brain regions. With improved culture conditions and equipment such as spinning mini bioreactor (Qian et al. 2018), the brain organoids can be cultured for a very long period that can mimic a more complete human brain developmental process at the transcriptome level (Marton et al. 2019) and epitranscriptome level (Yoon et al. 2017) as well as produce functional glial cells such as astrocytes (Sloan et al. 2017) and oligodendrocytes (Madhavan et al. 2018). Nevertheless, lacking correct cell arrangements and patterning (i.e. positional information), which is critical for the function of the specific tissue or organ, especially for those with a complicated spatial organization such as the brain (Wolpert 2016), has become one serious problem in the current organoid culture system. Until now this limitation has not been fully conquered. To solve this problem, it is meaningful to review how positional information is set up and interpreted during brain development in vivo.

### Spatial identity induction during brain development: a coordinate system of Wnt, BMP and Shh concentration gradients

#### BMP help specify the dorsal neural tube

BMPs are members of the TGF-β family, which play important roles during embryonic development, such as cell proliferation, differentiation, and migration (Shi and Massagué 2003; Guo and Wang 2009; Nelsen and Christian 2009; Bier and De Robertis 2015). More and more evidence indicate that BMPs participate in the formation of the neural tube and the dorsal cell fate specification (Hegarty et al. 2013; Le Dréau and Martí 2013).

For the precursor of the vertebrate central nervous system, the neural tube, its formation is quite complicated and can be traced back to a very early developmental stage before gastrulation. At the beginning, the dorsal organizer (Spemann’s organizer) secrets several BMP antagonists including noggin (Zimmerman et al. 1996), Chordin (Piccolo et al. 1996) and Follistatin (Fainsod et al. 1997), therefore inhibits the BMP expressing in the ectoderm surrounding the neural plate and helps form an early BMP concentration gradients. Such inhibition was proved to be necessary for the neuroectoderm specification and has been widely applied in the early stage of brain organoid differentiation (Smith and Harland 1992; Smith et al. 1993; Liu and Niswander 2005; Lancaster et al. 2013).

Later, the bending of the neural plate, invagination and further neural tube closure accompanied by dramatic cell movements have gradually transposed the initial pattern into the D-V axis (Nikolopoulou et al. 2017). During this process, the neural plate border, a broad area between the neural plate ectoderm and the nonneural ectoderm which has an intermediate level of BMP and contributes to the neural crest induction (Pla and Monsoro-Burq 2018), move to the dorsal side of the neural tube. After that, the roof plate, a dorsal midline region of the neural tube where the BMP is secreted, has gradually formed under medium level BMP induction (Nitzan et al. 2013).

**Fig. 2:**
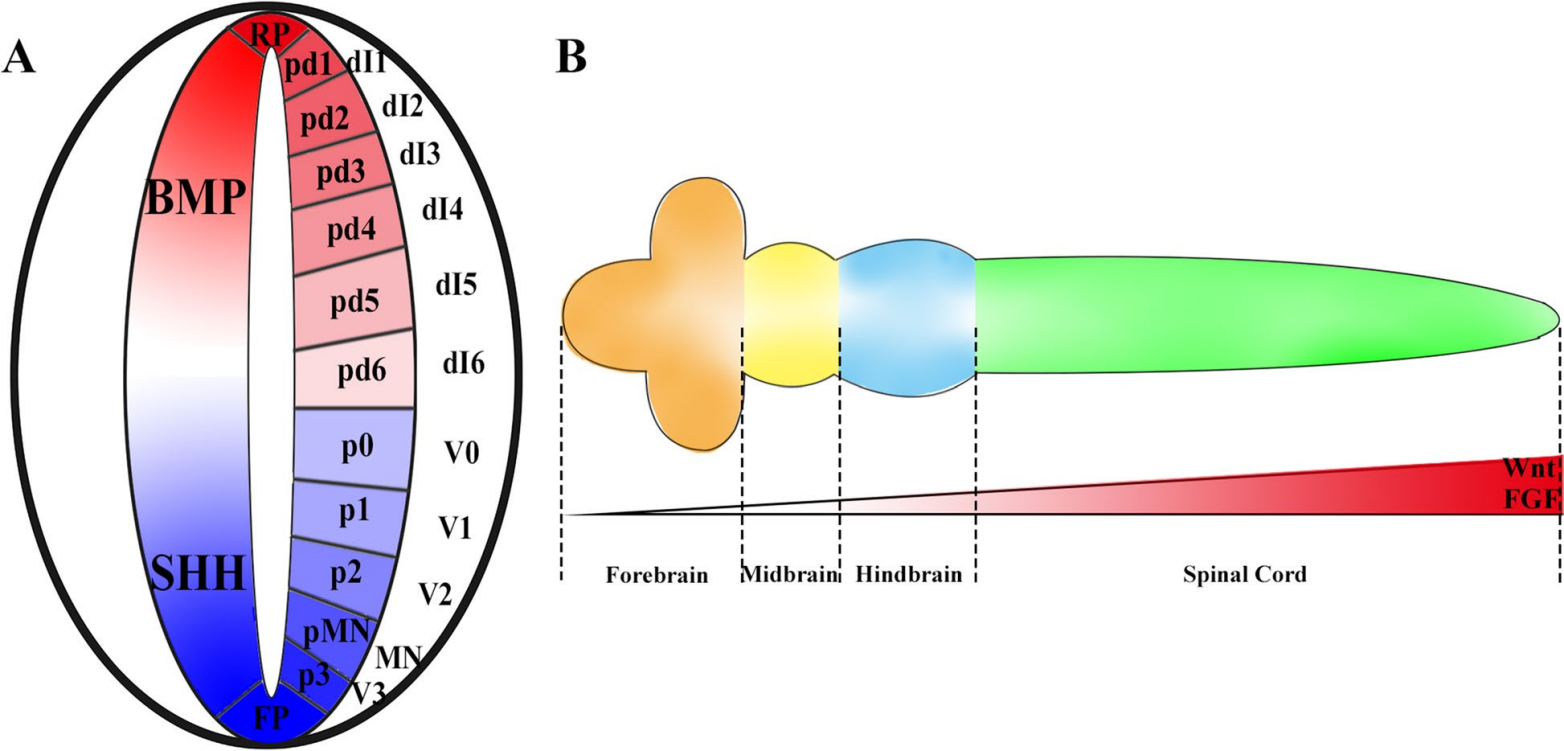
Concentration gradients of different morphogens pattern the neural development in vivo. **A** The dorsal–ventral patterning of the vertebrate neural tube. During the neural tube formation, the BMPs secret from the RP at the dorsal-most and the Shh secret from the FP at the ventral-most, forming two opposing gradients to pattern the neural tube. In response to the gradient signaling, the proliferative progenitor cells and the postmitotic neurons differentiate from the proliferative progenitor cells distributed in a specific order along the dorsal–ventral axis. The dorsal part contains six dorsal progenitor (dP) domains (dP1–dP6) and then differentiate into the dorsal interneuron (dI) populations (dI1–dI6). The ventral part contains five progenitor (p) domains (p0–p3 and the pMN) and differentiates into the ventral (V) interneurons (V0-V3, MN). **B** The anterior–posterior regionalization of the vertebrate nervous system. After the neural tube formation, the vertebrate neural tube can be further patterned along the anterior–posterior axis (rostro-caudal) axis into four parts (forebrain, midbrain, hindbrain and spinal cord) by a gradient of Wnt and FGF

There is much evidence that BMP secreted from the roof plate can act as a morphogen to specify the dorsal cell fate of the early neural tube and later spinal cord (Fig. 2A). In vivo, gain-of-function experiments suggest that different thresholds of BMP activities participate the setting of the expression boundaries of Pax6, which is the earliest sign to distinguish regional identity in neural tissue and induce different neural cell fate to form dI1 and dI2-3 neurons (Timmer et al. 2002). Double knockout of the BMP type 1 receptor, Bmpr1a and Bmpr1b, in the neural tube result in a complete loss of the DP1 domain and the DI2 classes interneuron population is profoundly reduced (Wine-Lee et al. 2004). Inhibition of the BMPs signaling pathway leads to a loss of the dI1 classes interneuron population and the dI2-4 classes interneuron population expanded by overexpression of noggin or knockdown of Smad4 (Chesnutt et al. 2004). Similarly, activation of the BMPs signaling pathway through overexpress both Bmpr1a and Bmpr1b leads to the dorsal and intermediate interneurons expansion and the loss of ventral interneurons (Timmer et al. 2002).

In the vertebrate neural tube patterning, different species have different BMP ligands, BMP4 and BMP7 are shown to be functional in chick while in mouse, BMP5, BMP6 and BMP7 are involved in the NT development (Liem et al. 1997; Andrews et al. 2017). There is also evidence showing that the dI3 was not effectively induced by the treatment with diluted BMP4-conditioned medium while the dI1 can be induced at BMP4-transfected COS cells in chicken neural tissue explants (Liem et al. 1997; Andrews et al. 2017). Therefore, some other mechanisms are proposed to explain the patterning of the neural tube.

Some studies found that not only the spatial distribution of the BMP signals, but temporal regulation is also required for such a complicated 3D organization. In the chick neural plate explant experiments, longer exposure to BMP4 leads to more dorsal progenitor identities. In vivo, inhibiting the BMPs signaling pathway at different stages results in different phenotypes. The early blockade results in a loss of the whole population of dI1-3 neurons, while later inhibition successively allowed the generation of dI3 and dI2 neurons (Tozer et al. 2013).

Besides, individual BMPs are responsible for the induction of particular neural fates (Lee and Jessell 1999). Loss of function mutations of Gdf7, another TGF-β family member, leads to the specific ablation of the Lhx2 + dI1A subpopulation in mice (Lee et al. 1998), while the other dI populations seem not to be impacted. Similarly, in the chick embryo, Bmp4 knockdown leads to the reduction of the dI1s, and Bmp7 knockout clearly decreases the number of dI1s, dI3s and dI5s (Le Dréau and Martí 2013). The mechanism can be explained by the dominant-negative type I Bmprs experiment which revealed that different BMPs can activate different type I receptors and regulate the cell cycle to participate in neuronal differentiation. The RP formation induced by the BMP6 in the mouse and BMP7 in the chick all function through the BmprIa. In both mouse and chick, the dp1 is induced by BMP4 and BMP7, through the BmprIa or BmprIb, but dI1 can only be induced by BMP4 but not BMP7. dI2 can be only induced by BMP4 in chick, but the dI3 can be induced by all BMP ligands. Interestingly, the dI4-dI6 cannot be induced by anyone BMPs (Andrews et al. 2017).

#### Shh specify the ventral neural tube

Shh is one of the most important Hedgehog (HH) glycoproteins in canonical HH signaling. It plays key roles in the various important developmental process like the development of vertebrate limbs, brain, muscle, gastrointestinal tract as well as the heart (Harfe et al. 2004; Hoffmann et al. 2009; Mao et al. 2010). During vertebrate neural tube development, Shh is secreted from the notochord and floor plate which are located at the ventral side of the neural tube (Jessell 2000). Shh expression is initiated first in the mesoderm during gastrulation, then expressing in the notochord, a solid rod of mesoderm forms at the midline of the embryo. In vertebrates, Dorsal–ventral patterning of the neural tube is largely dependent on notochord-derived Shh (Chamberlain et al. 2008; Yu et al. 2013). Inhibition of Shh maturation or secretion in mice would lead to a severe DV patterning failure in the neural tube (Zhang et al. 2001a, b).

There is also a lot of good evidence supporting Shh functions as a morphogen to specify cell fates in a concentration-dependent manner (Fig. 2A) (Dessaud et al. 2008). Patch1 is the direct receptor for Shh protein while the Hhip1 often serves as a regulator for Shh signaling. Normally, these inhibitory protein expressions on the membrane of neural progenitor cells can cause a cell-autonomous inhibition of Shh signaling through sequestration of the ligand and regulation of the degradation (Incardona et al. 2002). As Shh protein diffused distantly, the ligand availability is reduced due to the sequestration, leading to the weakening of the signal transduction (Chuang et al. 2003; Chuang and McMahon 1999; Goodrich et al. 1997; Jeong and McMahon 2005). When these ligand-dependent antagonisms (LDA) mediated by the Patch1 and Hhip1 is blocked, the fates of the neural progenitors seem to be transformed from the p0-p2 progenitors into the p3 and PMN progenitors which are located at the more ventral part of the neural tube (Jeong and McMahon 2005). In this case, the downstream cellular responses are subsequently changed to form a ventralized phenotype. In consistent with the sequestration, the endocytosis and degradation of these ligands are enhanced in the distant target field of Shh, which maintains the morphogen gradient together with the sequestration (Eldar et al. 2003).

Gas1 and Cdo are two stimulatory proteins on Shh signaling transduction. In wild-type embryos, the deletion of Gas1 also leads to a disruption in the ventral neural tube patterning. This defect is more severe in Shh deficiency embryos (Allen et al. 2007). Moreover, there is no floor plate, p3 or pMN domains in Gas1^−/−^; Cdo^−/−^ double mutant mouse embryos, suggesting a cooperative and synergistic role of these two proteins (Allen et al. 2007).

Gli is a key type of transcriptional regulator activated by Shh signaling that controls cell fates (Chen et al. 1999). In vertebrates, Gli-mediated graded Shh signaling relies on the regulation of three key members of the Gli family of zinc-finger-containing transcription regulators (Mahlapuu et al. 2001). Gli1 and Gli2 are mainly shown to function as Gli activators dependent on Shh, while Gli2 and Gli3 can be catalyzed into an inhibitory form to inhibit Shh and positive-related gene expression. The role of Gli1 is contentious since the Gli1 mouse mutants have normal spinal cord (Park et al. 2000) Different from Gli1 and Gli3, Gli2 possess both strong potentiating and inhibitory function, and its repressor activity cannot be regulated by Shh, providing an independent pathway to restrain Shh signal transduction (Aza-Blanc et al. 1997). In the current view, the predominant role of Gli2 is its activator activity. In Gli2–/– mouse embryos, the cell identities in the ventral neural tube, especially the floor plate, are severely disrupted (Ding et al. 1998; Park et al. 2000). For Gli3, it functions as a dose-dependent transcription repressor on Shh signaling. In Gli3 mouse mutants, a dorsalized phenotype is observed, supporting the inhibitory role of Gli3 on Shh (Persson et al. 2002). In addition, severe Shh phenotype can be partially rescued in the double mutants of Shh and Gli3 (Litingtung and Chiang 2000; Wijgerde et al. 2002). These experiments further confirmed the requirement of Shh signaling for normal dorsal–ventral patterning since intermixing of different neural progenitors can be identified (Bai et al. 2004; Litingtung and Chiang 2000; Wijgerde et al. 2002).

Similar to BMP and other morphogens, the accurate DV patterning is not only relied on the Shh concentration gradient, but also regulated by the stage and duration of the responsive cell exposed to the Shh ligands (Dessaud et al. 2007; Jeong and McMahon 2005; Stamataki et al. 2005). In 2007, Dessaud has proposed an excellent model called the ‘temporal adaptation’ model combining both the amount and duration of the Shh signaling (Dessaud et al. 2007). It is suggested that the sensitivity of the responsive cells will undergo a progressive decrease process. Initially, due to the high sensitivity to the Shh ligand, very low concentrations of Shh can activate adequate levels of Gli activity for gene expression and cell identity determination. With the development of the neural tube, desensitizing neural progenitor cells will induce a lower cellular response at the same Shh ligand concentration as that at the beginning. In this case, different patterning and cell fates can be induced at the different developmental stages under the same concentration (Dessaud et al. 2008). This model and the desensitization hypothesis are supported by the response of patch1. Upon the induction of the Shh, the responsive cells tend to express more inhibitory proteins like the Patch1 (Goodrich et al. 1996; Marigo and Tabin 1996). Therefore, to replenish these inhibitory interactions for normal signal transduction and cellular responses, there should be a higher concentration of Shh ligand (Dessaud et al. 2007).

#### FGF and Wnt signaling in the anterior–posterior regionalization of the vertebrate central nervous system

During the long-term development of the vertebrate central nervous system, the early neural plate can be gradually developed into four broad regions with distinct boundaries along its anterior–posterior axis – forebrain, midbrain, hindbrain and spinal cord. In contrast with the DV axis patterned by antiparallel BMP/Shh concentration gradient, the A-P axis specification seems to be more complicated by several discrete local organizers expressing the specific concentration of morphogens such as FGF and Wnt (Fig. 2B).

Wnt signals are representative growth factors that promote cell proliferation (Niehrs and Acebron 2012). Different from directly inducing cell proliferation, Wnt also controls the direction of cell proliferation through activating multiple downstream signalings and helps form the correct organized body pattern rather than amorphous organizations (Huang and Niehrs 2014; Wu et al. 2013).

In the neural development, a group of Wnt inhibitor, the Fizzled (FZD), is expressed in the anterior border of the neural plate (ANB), which becomes the anterior end of the neural tube after neural tube closure, therefore antagonize the Wnt8B expressing in the presumptive midbrain and posterior forebrain and help form the global gradient of Wnt signaling along the A-P axis (Houart et al. 1998). However, the role of such an inhibitory mechanism has not been elucidated in mammals yet. Someone argues that FGF8, which is also expressing at the ANB, contributes to the forebrain to midbrain patterning. In vitro treatment of the FGF8 in explants can mimic the anterior patterning (Shimamura and Rubenstein 1997). Also, knocking out FGF in the mouse and zebrafish leads to disruptions of the forebrain patterning (Meyers et al. 1998; Walshe and Mason 2003), supporting the role of FGF8 signaling in the A-P patterning. However, for the posteriorization of the neural development, Wnt plays more important roles rather than FGF (Woo and Fraser 1998).

Although there are still many arguments on whether Wnt and FGF pattern A-P neural patterning through concentration gradient (Green et al. 2015). Many early in vitro experiments do support the gradient hypothesis, for example, growing chick neural plate explants with different concentrations of Wnt3A and FGF8 could induce a different and restricted gene expression and cell fates along the A-P axis continually (Nordström et al. 2002). Also, zebrafish experiments further support such mechanism in vivo*.* Injections of different concentrations of the Wnt8a mRNA into the zebrafish embryos could induce the anterior hindbrain marker gbx1 while repress forebrain and midbrain genetic marker (e.g. otx2) expression in a dose-dependent manner (Rhinn et al. 2005).

### Application of the morphogen in the brain organoids: how to set positional information accurately

#### Morphogen has been widely used to direct cell fate specification in the stem cell-derived models

The success in organoid research was attributed to the early exploration of the morphogens as microenvironmental cues in many in vitro models, especially the ESCs and later the iPSCs. Early in 1981, Martin and his colleagues have already obtained the mouse ESCs from the inner cell mass of a blastocyst. The human ESCs were later successfully derived in 1998 (Thomson et al. 1998). The Yamanaka factors help reprogram the somatic cells into a pluripotent state called iPSCs (Takahashi and Yamanaka 2006), providing an opportunity to generate a large number of pluripotent stem cells in vitro. These models offer many insights into developmental biology and help elucidate the role of different morphogens in directing cell differentiation into specific cell types in vitro*.* For example, BMP signals transposed to the serum-free culture medium of ESCs could promote non-neural cell differentiation (Wiles and Johansson 1999), and it is achieved by activating the helix-loop-helix transcriptional inhibitor, Id proteins (Ying et al. 2003). In contrast, blocking BMP using its inhibitor Noggin could induce neuronal differentiation in the ESCs (Gratsch et al. 2002). Therefore, the initial steps of most current methods generating 3D brain organoids all require different BMP signaling inhibitors such as SB43154, LDN193189, dorsomorphin and A-83 to induce neuroectoderm cell fates (Muguruma et al. 2015; Jo et al. 2016; Qian et al. 2016).

Besides BMP, the addition of other morphogens such as Nodal/activin also helps direct cell differentiation from a pluripotent stem cell. An intractable problem for researching ESCs at the beginning is that it is hard to define primitive and definitive endoderm since their gene expression has a large overlapping. However, the addition of Nodal/activin, a vital factor in mesendoderm specification (Schier 2003), could block neuronal differentiation and induce the expression of the definitive endoderm-specific gene *brachyury* in a dose-dependent manner (Kubo et al. 2004)*.* Therefore, different doses of Nodal/activin were then used to induce mesendoderm-derived structures such as heart, muscle, lung, intestine and so on in vitro (Kattman et al. 2011; Múnera et al. 2017).

Wnts also play specific roles in cell fate specification. Adding Wnt1/Wnt3a to the ESC culture media could restrict neural differentiation of the ESC and enhance the *brachyury* induction (Watanabe et al. 2005), while blocking Wnt using its inhibitor sFRP2 could promote neuroectoderm induction (Aubert et al. 2002).

The role of FGF in cell fate specification is more complicated as it comprises three main downstream signalings (Mossahebi-Mohammadi et al. 2020). On the one hand, FGF assists the formation of primitive endoderm and promote hematopoietic commitment (Faloon et al. 2000; Li et al. 2004). On the other hand, FGF could promote neuroectoderm differentiation using a monolayer differentiation culture (Ying et al. 2003). Overall, these in vitro stem cell models have created an excellent opportunity to help verify the role of different morphogens during cell fate specification and made it possible to generate cell types of interest in vitro.

#### The addition of the morphogen help resembles more precise regions in the brain organoids

Over the past two decades, multiple studies have improved the morphology of the aggregates and the organoids with different bioengineered strategies to achieve better reproducibility (Fedorchak et al. 2021). However, those methods relying on self-organization could not be able to recapitulate more precise structures of the brain. In this case, the application of the morphogen should receive more consideration.

With sufficient comprehension of the role of different morphogens at different developmental stages both in vivo and in vitro. Many attempts using 3D culture systems together with different morphogen addition were done to recapitulate a more precise structure of the brain in vitro.

3D neuroepithelial cyst model is a simple approach to uniformly generate neural plate progenitors that resemble dorsal neural tubes and responsive to different morphogens such as Shh (Meinhardt et al. 2014). RA supplement could adjust the anterior–posterior potential of these cysts. Moreover, under the appropriate concentration of RA, the localized floor plate could be induced and partially reproduce the dorsal–ventral patterning (Meinhardt et al. 2014), offering an ideal spinal cord model to study morphogen.

SFEBq is another mature system applying self-organizing theory to generate complicated and well-organized 3D structures created by Yoshiki Sasai Lab (Eiraku et al. 2008). Using such self-organizing strategies with specific improvements, Sasai’s group has made great progress in generating 3D brain structures such as optic cup, neocortex, cerebellum, hippocampus and pituitary gland in the past few years (Kadoshima et al. 2013; Muguruma et al. 2015; Ozone et al. 2016; Sakaguchi et al. 2015). In these studies, the application of the appropriate morphogen could provide necessary positional information that refines the substructure of the brain. For example, the addition of FGF19 could promote the spontaneous generation of hindbrain neural-tube-like structures with dorsal–ventral patterning (Muguruma et al. 2015). A sequential supplement of SDF1 could further generate a more refined rhombic-lip-like structure that better recapitulate the cerebellum (Muguruma et al. 2015).

#### Refined patterning in the brain organoids relies on appropriate concentration gradients of the morphogen

The sequential addition of specific morphogens together with improving 3D culture systems do help recapitulate several precise brain structures. However, these methods all directly supply the morphogen through the medium, the enriched morphogens and other intrinsic signals surrounding the organoids during the 3D culture may compromise the construction of the morphogen concentration gradient, therefore disrupt the positional information (Corrò et al. 2020). To reproduce the concentration gradient in the brain organoid culture system, there are mainly three bioengineering strategies so far.

**Fig. 3:**
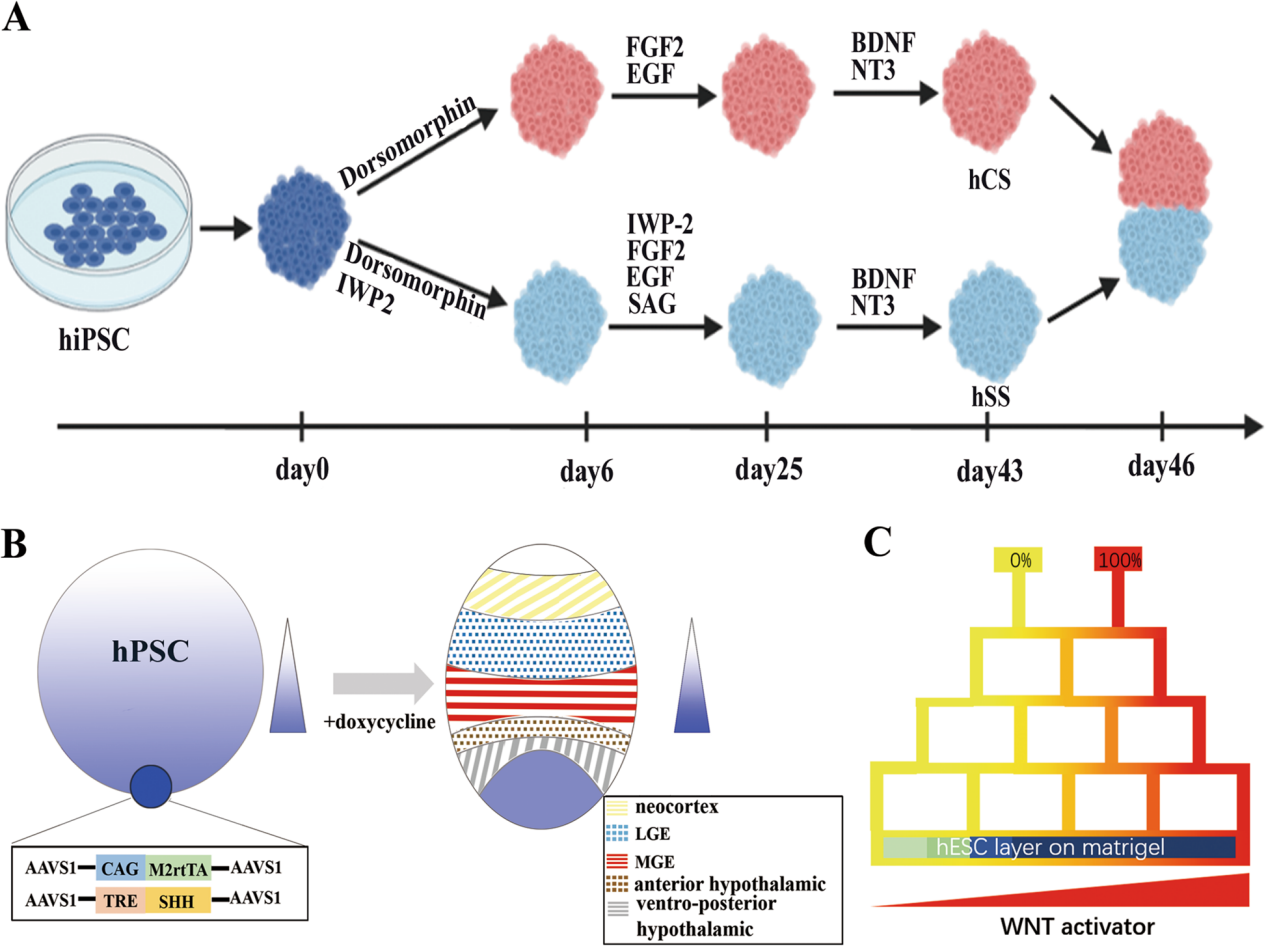
The bioengineered strategies to produce morphogen concentration gradient in the brain organoid culture. **A** The robust protocol to generate 3D dorsal-patterned and ventral-patterned forebrain organoids by Sergiu Pasca (Birey et al. 2017). These formed organoids could be further assembled to generate a bigger forebrain organoid with separated dorsal ventral brain regions. **B** Construct a forebrain organoid. A doxycycline-inducible Shh human H9 cell line was generated and aggregated to form an organizer center for another organoid to provide an Shh concentration gradient. After doxycycline induction, a patterned forebrain organoid was generated, the organoid recapitulates the topography of the in vivo forebrain, and have formed the neocortical, lateral ganglionic eminence (LGE), medial ganglionic eminence (MGE), anterior hypothalamic and ventral-posterior hypothalamic at the appropriate position (Cederquist et al. 2019). **C** A scheme of the microfluidic system which can culture several organoids exposed to different concentrations of the Wnt signals

First, the generation of assembloids, a co-culture system of different kinds of organoids (Andersen et al. 2020), provides the possibility to integrate different region-specific brain organoids to form more complete and functional brain organoids (Fig. 3A). Currently, the differentiation protocol of different regional brain organoids through sequential addition of different signals is highly reproducible and the relevant differentiation kit has been produced (Birey et al. 2017). For example, temporal-controlled addition of FGF19 and SDF1 could help the organoid differentiate into a cerebellum-like cell fate (Muguruma et al. 2015). Additionally, the addition of the Wnt antagonist IWP-2 could help separate the dorsal and ventral fates of the forebrain (Birey et al. 2017). Until now, these region-specific brain organoids could well recapitulate the molecular and cellular features of various regions of the human brain including the forebrain, midbrain, hypothalamus, hippocampus and so on (Jo et al. 2016; Qian et al. 2016; Sakaguchi et al. 2015). After generating the assembloids based on the physiological positions of different organoids, different signals like morphogens could be secreted from the cells at the corresponding positions and diffused to form appropriate concentration gradients.

Second, the 3D co-culture strategy could imitate the organizer to reproduce a more complete self-organization process (Fig. 3B). Cederquist et al. have generated a small cluster of hPSCs expressing Shh proteins and co-cultured it with bigger clusters of normal hPSCs to generate a concentration gradient of Shh (Cederquist et al. 2019). This co-culture system has successfully mimicked a forebrain organoid with Shh-dependent ventral patterning. This strategy could also be expanded to other organizers expressing different morphogens such as BMP and Wnt. Alternatively, many biological engineers also combined hydrogel culture system with the morphogen-soaked beads and use this system to model the role of local organizer. Microbeads with different concentrations of morphogens can be delivered to a specific region and induce the asymmetrical stem cell division at the single-cell level (Habib et al. 2013). In 2020, Ben-Reuven and Reiner have created a brain-on-chip protocol to research the BMP4 and Wnt concentration gradient together. In this system, brain organoids exposed to different concentrations of the morphogen can be induced to express markers for different brain regions (e.g. telencephalon and medial pallium, dorsal and ventral midbrain, isthmus) (Ben-Reuven and Reiner 2020). Compared to the live co-culture system, although these beads are not real cell clusters and cannot proliferate and migrate, due to its convenient manipulation which can study multiple organizers simultaneously, it still has adequate significance to the research of morphogen gradient application in the 3D brain organoid culture, especially for the beginning stage at present.

Third, a powerful approach for creating complex biomolecular gradients is the use of the microfluidic system (Fig. 3C), which can generate spatiotemporally adjustable morphogen gradients. These devices provide adequate confidence to alternatively trigger the self-organization of specific cells in a correct manner. Due to the limitation of the current techniques, it is often applied in the 2D culture system, in a recent study, through reproducing the anti-parallel gradients of Shh and BMP, together with the other two related signaling-Wnt and RA, different types of neural cells can be induced in different spaces from mouse embryonic stem cell (Demers et al. 2016). Later such a system that controls the morphogen gradients spatiotemporally also successfully recapitulates the different axially arranged domains in 2D human PSC cultures (Manfrin et al. 2019).

### Discussion & future perspectives

With such a short history of less than 10 years, brain organoid technology has brought us too much surprise to strengthen our understanding of human brain development and the relevant brain diseases. Many problems appear at the early stage of this technology such as lack of reproducibility, lack of cell-type specificity, heterogeneity and uncontrolled size have been largely improved in these years with the advance in engineering and stem cell biology. For example, the mixing of extracellular matrix and other additives with the biomaterial could produce a cultural environment that is more similar to the actual human brain and generate a high-reproducible brain organoid (Yin et al. 2016). The micro-structured scaffolds can help control 3D brain organoid differentiation (Lancaster et al. 2017). Also, 3D bioprinting technology could help to precisely sort prepatterned progenitors to control the size and content of the initial culture medium (Vijayavenkataraman et al. 2018). Under such improved conditions, the genetic engineered system and high-throughput drug screening in the patient-derived brain organoids have largely expanded the clinical study. This technology successfully bridges the gap between 2D cell models and in vivo animal models for human brain research.

As we discussed in this review, lacking accurate positional information is a nonnegligible problem in organoid research. In 2013, Sasai has already raised the idea that providing 3D self-organization cell cultures with positional information corresponding to morphogens presented during embryogenesis is the next generation of organogenesis (Sasai 2013). Thankfully, more and more deep-going research on morphogen gradient and advancing bioengineering techniques raised in recent years (Noor et al. 2019), offering an optimistic future to overcome this challenge and improve the similarity between the human brain and the brain organoids. Establishing correct positional information may also promote functional research in brain development and relevant diseases. Since cell–cell interactions are important for brain development and functions, early disordered cell arrangement might impact the maturation and function of the brain. As current protocols for the 3D brain organoid development can only mimic the molecular and cellular level of early human brain development (around second trimester) (Qian et al. 2016), therefore limit the research of neurodegeneration and some age-related diseases such as Alzheimer’s diseases. Setting up the correct positional information might also help to induce more matured cell types and generate a more complete brain with some of the functions.

However, there are still many obstacles that might not be solved only by providing spatial cues. For example, the absence of a vascular system restricts the growth and maturation of the organoid due to the limited oxygen and nutrients diffused from the culture medium, especially for the deep cells inside. Luckily, there are many successful attempts to establish vascular systems in brain organoids. Besides transplantation to the adult mouse brain to provide a vascularized physiological environment (Mansour et al. 2018; Wimmer et al. 2019), ectopically expression of ETV2 could help induce endothelium and form a vascular-like network in the brain organoid in vitro (Cakir et al. 2019). The assembloid strategies also work for establishing vasculature in the brain organoids, co-culture brain organoids with endothelial cells (Pham et al. 2018) and/or mesenchymal stem cells (Song et al. 2019) could partially recapitulate the structure and function of vasculature and blood–brain barrier.

Also, such assembloid technology could be applied to supplement other missing non-neural cells in the human brain such as microglia and endothelial cells. Alternatively, a recent study reported that the organoid without SMAD inhibition at the early stage could contain several mesodermal progenitors that are able to differentiate into mature microglia (Ormel et al. 2018). It is also possible to culture a single brain organoid with other non-neural cells through precise differentiating regulation using advanced bioengineering strategies. Furthermore, 3D printing technology may offer more opportunities for bioprinting required tissues such as vasculature in specific scales (Noor et al. 2019).

Moreover, bioengineering research also promotes functional reproduction in the organoid. Early in this century, scientists have already found that geometry and patterning could improve the activity of cultured neuron networks (Nam et al. 2004; Wheeler and Brewer 2010). Such role of neural patterning has also been gradually applied in the 3D culture system, a representative research uses the assembloid strategy to create functional cortical-motor neural circuits (Andersen et al. 2020). Besides, biomechanical signals should receive more attention in future organoid research. Several studies have already pointed out the importance of membrane tension in cell pluripotency and cell differentiation (Bergert et al. 2021). Adding these factors in the bioengineered systems may help construct a completer and more mature organoid.

## Conclusions

Overall, the brain organoid is a cutting-edge technology in the research of the human brain. Combining advanced research strategies and techniques, we could have an opportunity to better understand the human brain from broad perspectives not only limited to genetic, molecular and cellular level but also biomechanics. Establishing appropriate morphogen gradients have been suggested as a promising and effective approach to provide the positional information during human neural organoid culture, which largely improve the patterning and the maturation of the organoids. Although there are still many challenges, we strongly believe after replenishing those missing puzzles, this technology will bring us more surprise.

## Data Availability

Not applicable.

## Abbreviations

SFEBq: Embryoid-like aggregate culture with quick reaggregation
ESC: Embryonic stem cell
vRGCs: Ventricular radial glial cells
oRGCs: Outer radial glial cells
iPSCs: Induced pluripotent stem cells
PI: Positional information
RA: Retinoic acid
FGF: Fibroblast Growth Factors
GDF: Growth Differentiation Factors
Wnt: Wingless-Int
BMPs: Bone morphogenetic proteins
Shh: Sonic Hedgehog
HH: Hedgehog
LDA: Ligand-dependent antagonisms
FZD: Fizzled
ANB: Anterior border of the neural plate
dP: Dorsal progenitor
dI: Dorsal interneuron
LGE: Lateral ganglionic eminence
MGE: Medial ganglionic eminence
